# An Active Three-in-One Teaching Approach Integrating Article Analysis, SPSS Simulation, and Lectures in Biostatistics Education for Medical Students

**DOI:** 10.7759/cureus.102038

**Published:** 2026-01-21

**Authors:** Néstor Israel Quinapanta Castro, Andres F Orbea

**Affiliations:** 1 Faculty of Philosophy and Letters, University of Buenos Aires, Buenos Aires, ARG; 2 Department of Research, Regional Autonomous University of the Andes (UNIANDES), Ambato, ECU; 3 Department of Medicine, Regional Autonomous University of the Andes (UNIANDES), Riobamba, ECU

**Keywords:** applied biostatistics, education and training, medical school education, teaching and research, teaching biostatistics

## Abstract

Introduction: Medical students recognized the usefulness of biostatistics in clinical practice but also perceived it as a complex, impractical subject. This study evaluated the effect of the three-in-one (3-in-1) active teaching method on academic performance in biostatistics, as well as on student satisfaction and perceived understanding, among second- and ninth-semester medical students using a comparative educational design stratified by academic level.

Methodology: An observational, prospective, post-educational intervention study was conducted with stratification by academic level (second and ninth semesters). Random assignment within each stratum was performed using SPSS version 29 (IBM Corp., Armonk, NY). The study population consisted of students enrolled in the medical program at the Regional Autonomous University of the Andes during the 2025 academic year. Students who attended at least 80% of the sessions and provided informed consent were included; those who could not participate due to limitations or withdrew their consent were excluded. The sample was divided into two groups: an intervention group that received the “3-in-1” active method (article analysis, SPSS simulation, and lecture) and a control group that continued with the traditional methodology. Academic performance was measured using an 11-question multiple-choice test (Médico Interno Residente, MIR), while attitudes, mastery, and satisfaction were assessed using a Likert scale. The statistical analysis included descriptive statistics, Student's t-test, Mann-Whitney U test, and chi-square test, all with a significance level of p < 0.05.

Results: The sociodemographic characteristics of the groups were comparable, with no significant differences in gender, cohabitation status, ethnicity, geographical origin, or academic history. Only age differed between the groups (p = 0.016). Regarding academic performance, the intervention group had a mean score of 13.13, while the control group had a mean score of 10.92, a statistically significant difference (t = −2.346; p = 0.023). Satisfaction with the teaching method was significantly higher in the intervention group (U = 379.5; p = 0.016). Likewise, the "3-in-1" group had a higher perceived mastery of the content (7.74 vs. 5.58; p < 0.001). There were no significant differences in the comparison by academic semester (p = 0.511), suggesting that the level of training did not influence the scores obtained.

Conclusion: Compared to the traditional methodology, the active “3-in-1” methodology was associated with higher academic performance, greater student satisfaction, and a better perception of content mastery. These results suggest the importance of using active and applied pedagogical strategies when teaching biostatistics to medical students.

## Introduction

Biostatistics provides the theoretical basis for deriving knowledge from variable and uncertain data. This makes biostatistics essential for empirical research in public health and clinical medicine [1]. Biostatistics is applied at every stage of the research process, from design to analysis and interpretation of results. Teaching biostatistics is crucial for maintaining the quality of biomedical research and evidence-based medicine (EBM), both of which are vital for the health and well-being of the global population [2].

The use of statistics in medical practice has increased significantly because statistics are a fundamental tool for supporting diagnoses and guiding therapeutic decisions. Proper application of statistics allows clinicians to interpret disease progression and treatment based on data from patients with similar characteristics. However, many physicians still have a poor grasp of statistical concepts, which hinders their ability to correctly evaluate scientific evidence [3].

In recent years, the quality of research results published in scientific articles has declined due to errors in statistical analysis and inadequate reporting [4]. Several reviews [5,6] have also shown that a significant amount of waste and inefficiency in health research stems from the misuse of statistical methods, which is often associated with inadequate methodological training. The growth of EBM and digital technologies, as well as the extensive use of mathematical methods in health, has greatly increased the demand for professional competence [7].

However, students often struggle to integrate theoretical statistical concepts with real-world practice, which can hinder their ability to apply this knowledge in future careers [8]. Biostatistics is an essential component of professional training, yet it is often underestimated by health science students due to its apparent remoteness from clinical practice. Insufficient statistical training becomes evident after graduation in professional practice and in interpreting scientific articles [9]. A limited understanding of statistics can lead to flawed research methods and contribute to the reproducibility crisis [10,11].

Only 68.0% of medical students have a basic understanding of biostatistics, and only 36.5%, 33.0%, and 27.4%, respectively, have knowledge of parametric and nonparametric hypothesis testing and statistical software packages [12]. Furthermore, only 60% of students consider their acquired biostatistical knowledge to be useful for their careers. Additionally, 57.7% of students indicated that the lack of practical exercises made the course more difficult. Only 35.1% of students positively assessed the importance of biostatistics modules [13].

Although there are several teaching methods, students consider practical biostatistics teaching to reduce stress associated with learning and exams (p < 0.001), improve knowledge levels (p < 0.001), and increase satisfaction upon passing (p < 0.001) [4]. One successful intervention in biostatistics teaching was a study conducted at the Faculty of Medicine at Muhammadiyah University in Malang (FMUMM). The results showed that students who worked with the biostatistics center obtained significantly higher biostatistics knowledge scores (p = 0.0000) [8].

Medical statistics teaching at the undergraduate level is oriented toward the EBM paradigm, combining theory and practice [14]. Therefore, there is a need to reorient the teaching of biostatistics in medical education toward an integrated, applied, and clinically relevant approach [15]. Using active methodologies, such as small-group work, and incorporating technological and computational tools are considered essential for developing analytical and data-interpretation skills in clinical contexts.

This study aims to compare the effects of the three-in-one (3-in-1) active teaching method and traditional instruction on academic performance in biostatistics (the primary outcome), as well as student satisfaction and perceived understanding of the content (the secondary outcomes), among second- and ninth-semester medical students. The study uses a non-experimental, comparative educational design, which is stratified by academic level.

## Materials and methods

Design

This was an observational, prospective, post-educational intervention study with a comparative approach. It was based on data derived from an internal institutional study that was conducted to support the orientation and adjustment to the new academic curriculum at the Faculty of Medicine at the Regional Autonomous University of the Andes (UNIANDES). It was conducted among undergraduate medical students. Stratification was carried out according to academic semester (the second and ninth) to control for variability associated with differences in the curriculum, cognitive ability, and academic experience across educational levels. This ensured greater internal homogeneity within each academic group and reduced bias derived from differences in prior knowledge.

Study population

The study population consisted of second- and ninth-semester medical students enrolled at UNIANDES during the 2025 academic year. Inclusion criteria included being officially enrolled in the corresponding semester, attending at least 80% of biostatistics course sessions during the semester, and providing written informed consent to participate in the study. Students who failed to attend their scheduled appointment were excluded.

To ensure adequate representation of the semester variable and control it, the sample was stratified by academic level (second and ninth semesters). Within each stratum, simple random assignment was performed using the statistical software SPSS version 29 (IBM Corp., Armonk, NY) to form the intervention and control groups. This methodology balanced the distribution of participants by semester and ensured comparability between groups, thereby optimizing the study's internal validity.

**Figure 1:**
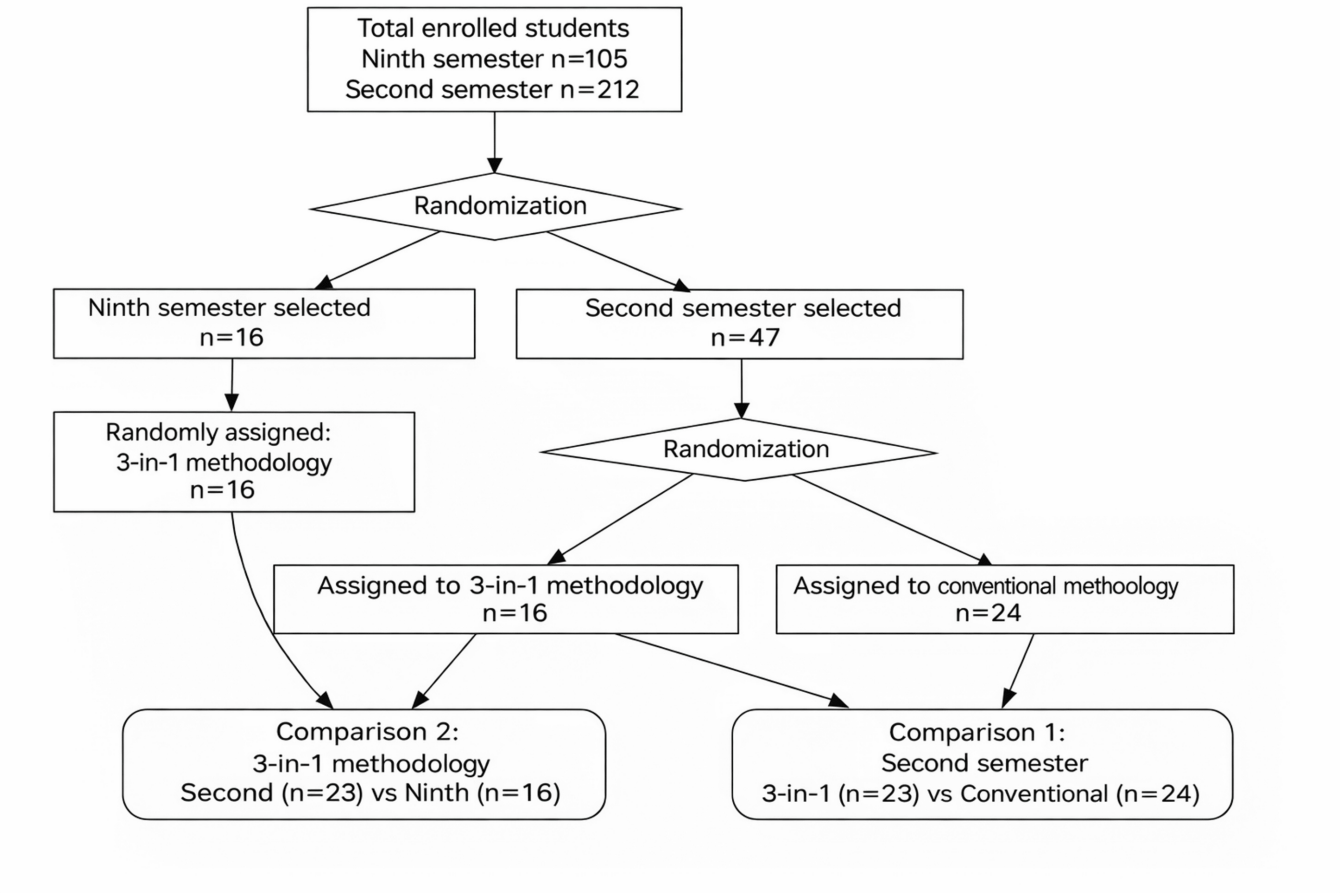
Student selection flowchart.

A stratified, randomized sample was selected and assigned to two groups: an intervention group and a control group (Figure 1). The intervention group received instruction using an active "3-in-1" approach that integrated reading and analyzing scientific articles, SPSS simulations with databases, and lectures. The control group continued with the traditional methodology based on conventional lectures.

Instruments

A standardized, multiple-choice test consisting of 11 questions was used to collect data, following the Médico Interno Residente (MIR) methodology (Appendix). The questionnaire was designed by academic peers, specifically biostatistics instructors from the medical program. Its content validity was assessed by three experts in biostatistics and research methodology, including a physician epidemiologist who serves as head of the research department and two research faculty members from the medical school. Additionally, the instrument was validated and formally approved by the director of the medical program.

Procedure

Immediately after the intervention, all students participated in an academic assessment designed to measure their mastery of biostatistics content. The assessment was a standardized, multiple-choice test developed using the MIR methodology. It evaluated fundamental knowledge, interpretation of statistical tests, and application of concepts in practical scenarios. Biostatistics teachers from the medicine program developed the question bank, and three experts in research methodology reviewed it to ensure apparent validity, pedagogical relevance, and alignment with the educational level of second- and ninth-semester students. Likert scales were used to complement the evaluation of the educational process by measuring satisfaction with the teaching method and self-perception of content mastery. The control group received traditional instruction, which was lecture-based, while the intervention group participated in an active "3-in-1" approach that integrated the analysis of scientific articles, practical simulations in SPSS, and a supplementary lecture.

The control group received traditional instruction in the form of a single 200-minute lecture. This focused on the systematic presentation of theoretical content, accompanied by illustrative examples and guided problem-solving activities led by the teacher.

The intervention group participated in the active "3-in-1" approach, which was delivered over the course of a 200-minute face-to-face session. This began with a 60-minute introductory lecture, which aimed to establish the necessary conceptual foundations, standardize prior knowledge, and contextualize statistical content. This was followed by a guided critical analysis of scientific articles (70 minutes), during which students identified study designs and statistical tests, as well as the correct interpretation of results. Finally, practical simulations were carried out using SPSS software (70 minutes) to enable the direct application of concepts through database analysis, selection of statistical tests, and interpretation of program outputs.

At the end of the session, both groups took the same objective test and subjective evaluation under equivalent and controlled conditions. This allowed for a standardized comparison of the impact of the "3-in-1" method on the primary and secondary outcomes of the study.

Primary and secondary outcomes

The primary outcome was the score obtained by students in the biostatistics academic activity, measured using a test designed to assess mastery of the taught content. Secondary outcomes included student satisfaction with the teaching method and self-perception of mastery of the class content, which was collected using Likert scales administered immediately after the session. Additionally, a comparative analysis by academic semester was conducted to explore potential differences related to the level of education.

Statistical analysis

IBM SPSS Statistics version 29 was used to perform a descriptive analysis of the sociodemographic and academic variables. Quantitative variables were analyzed using a Student's t-test for independent samples, after confirming normality and homogeneity of variances. Ordinal variables were examined using a Mann-Whitney U test. Nominal variables were analyzed using a chi-square test, and odds ratios (ORs) were calculated for dichotomous variables. The statistical significance level was set at p < 0.05.

Bioethical considerations

This study included medical students. Written informed consent was obtained from all participants prior to their inclusion in the study, and no data that could identify patients were collected. As the study was conducted as part of an educational evaluation and complied with institutional guidelines for student research, ethical approval for human participation was not required.

Presentation of preliminary findings

The preliminary results of the study were presented on October 16, 2025, at the Second International Conference on Family Health: Research and Outreach. This event was organized by the Faculty of Health Sciences at San Martín University (Bogotá Campus) through its Medicine Program and Research Coordination.

## Results

Second-semester students

Baseline Characteristics

A comparison of the baseline characteristics revealed that most of the categorical variables were distributed evenly across the groups with no statistically significant differences in gender (p = 0.474), household composition (p = 0.098), ethnicity (p = 0.354), geographic origin (p = 0.223), or history of semester loss (p = 0.932), as shown in Table 1. The total sample was predominantly female, Mestizo, and from the Sierra region. Additionally, a higher proportion of students living alone was observed in the "3-in-1" group, though this difference was not statistically significant. Of the continuous variables, only age showed a significant difference between groups (p = 0.016), with a higher mean in the control group. The number of semesters repeated remained low and similar in both groups, with no statistical difference observed (p = 0.713).

**Table 1:** Baseline characteristics of the participants. * Chi-square test; ** Student’s t test.

Variable	Categories	Control, n (%)/Mean ± SD	“3-in-1”, n (%)/Mean ± SD	Total, n (%)/Mean ± SD	Test (χ²/t)	p-value
Sex	Male	8 (33.3)	10 (43.5)	18 (38.3)	0.512*	0.474
	Female	16 (66.7)	13 (56.5)	29 (61.7)		
Living arrangement	With parents	9 (37.5)	8 (34.8)	17 (36.2)	4.665*	0.098
	Alone	11 (45.8)	15 (65.2)	26 (55.3)		
	Relatives	4 (16.7)	0 (0.0)	4 (8.5)		
Ethnicity	Afro-Ecuadorian	0 (0.0)	1 (4.3)	1 (2.1)	2.075*	0.354
	Indigenous	1 (4.2)	3 (13.0)	4 (8.5)		
	Mestizo	22 (91.7)	20 (82.6)	42 (89.4)		
Geographic origin	Highlands	21 (87.5)	20 (87.0)	41 (87.2)	3.004*	0.223
	Coast	2 (8.3)	0 (0.0)	2 (4.3)		
	Amazon region	1 (4.2)	3 (13.0)	4 (8.5)		
Semester failed	No	18 (75.0)	17 (73.9)	35 (74.5)	0.007*	0.932
	Yes	6 (25.0)	6 (26.1)	12 (25.5)		
Age (years)		20.46 ± 1.38	19.57 ± 1.04	20.02 ± 1.29	2.497**	0.016
Repeated semesters		0.25 ± 0.44	0.30 ± 0.56	0.28 ± 0.49	−0.371**	0.713

Knowledge Test Score ("3-in-1" Versus Control)

**Figure 2:**
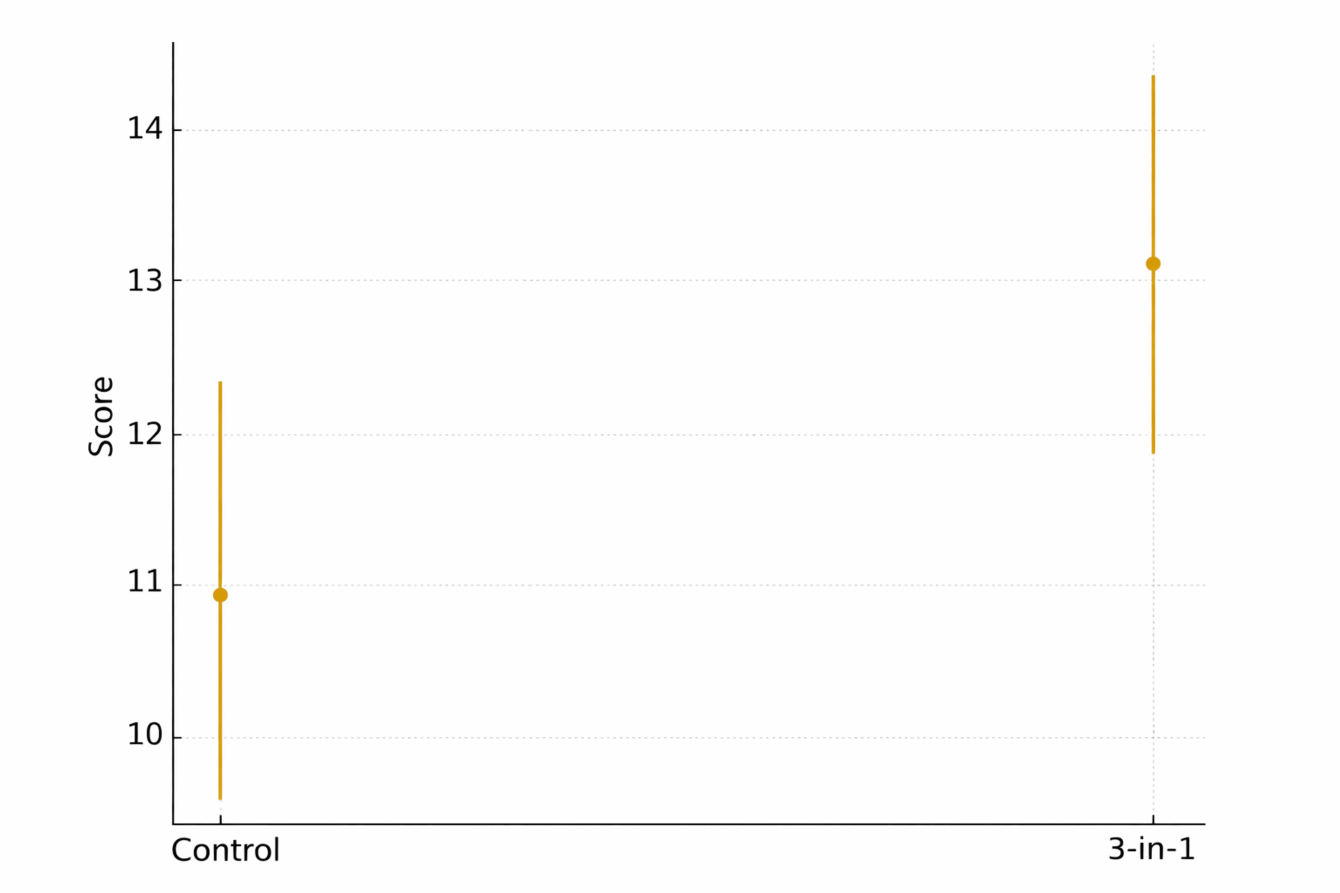
Comparing the mean and 95% CI biostatistics knowledge test scores of the control and “3-in-1” groups.

The control group obtained an average score of 10.92 (95% CI: 9.49 to 12.35), while the “3-in-1” intervention group achieved an average score of 13.13 (95% CI: 11.81 to 14.46), as shown in Figure 2. This indicates that the intervention group performed better on average.

This analysis compared the scores obtained by the intervention and control groups using a Student's t-test for independent samples (Table 2). The control group (n = 24) achieved an average score of 10.92 ± 3.387 (mean ± SD), while the "3-in-1" intervention group (n = 23) achieved an average score of 13.13 ± 3.065. Levene's test indicated homogeneity of variances (F = 0.786; p = 0.380), thus assuming equality of variances for the contrast. The t-test results revealed a significant difference between the groups (t = -2.346, p = 0.023), with an average difference of -2.214 (95% CI: -4.114 to -0.314). These results suggest that the "3-in-1" intervention positively impacted the mean score compared to the control group.

**Table 2:** Student's t-test: comparison between the control group and the "3-in-1" group.

Group	N	Mean ± SD	t	Sig.	Mean difference	95% CI, lower	95% CI, upper
Control	24	10.92 ± 3.39	-2.346	0.023	-2.214	-4.114	-0.314
Intervention (“3-in-1”)	23	13.13 ± 3.06					

The recoded score was dichotomized based on whether participants scored less than or equal to 11.2 points (70% of the total score). This threshold was chosen because it corresponds to the minimum passing grade set out in the internal regulations of the UNIANDES, and because it aligns with the academic regulations of Ecuador's Higher Education Council (CES). Analysis using Pearson's chi-square test revealed a significant association between the intervention/control group and passing the knowledge test (χ² = 6.88; df = 1; p = 0.009). From an epidemiological perspective, the odds ratio (OR) was 5.61 (95% CI: 1.46-21.53), indicating that participants in the control group were 5.6 times more likely to score below 11.2 points than those in the intervention group.

Satisfaction With the Teaching Method

**Figure 3:**
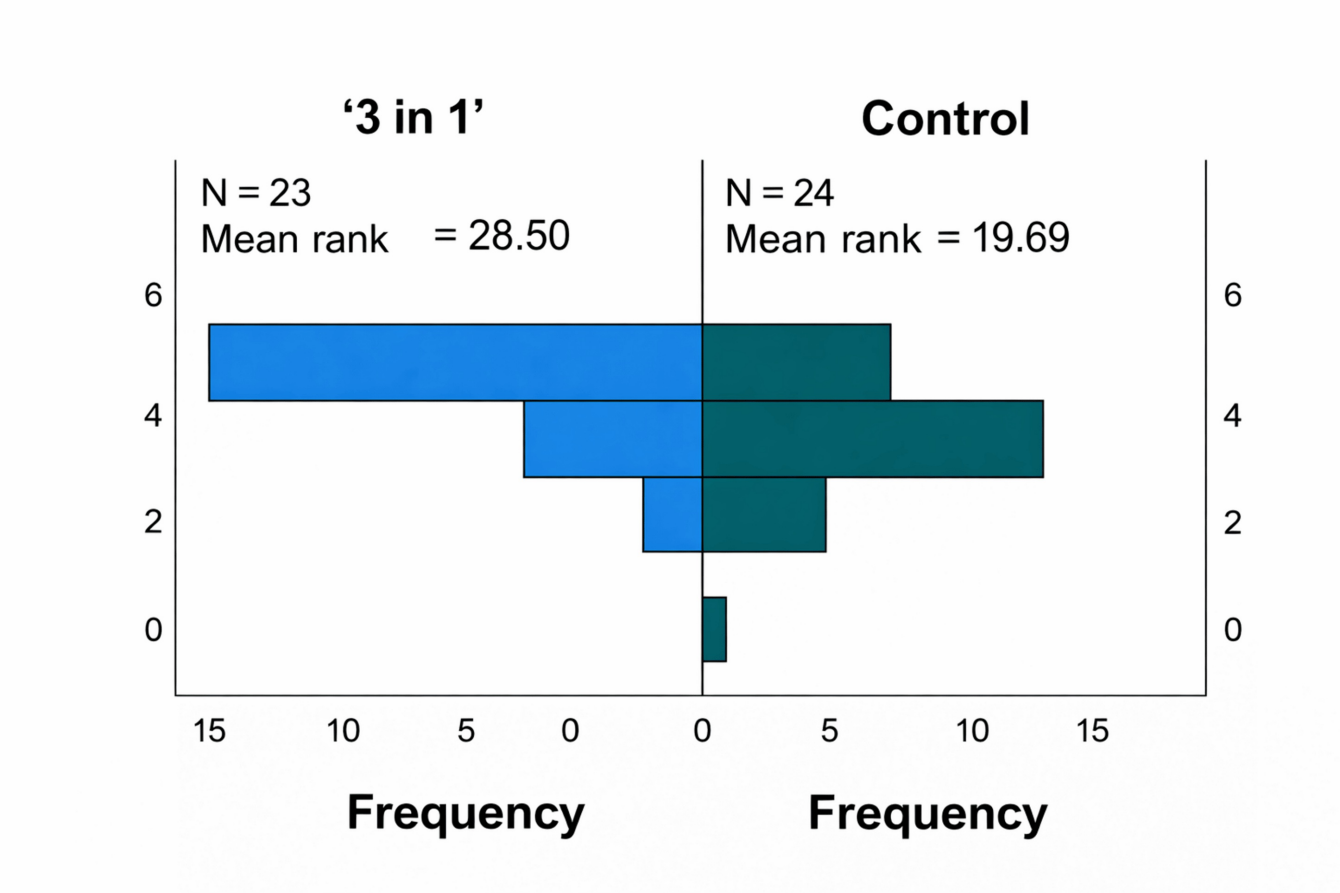
The Mann-Whitney U test: satisfaction with the teaching method.

The "3-in-1" method produced higher average scores and a greater concentration of high satisfaction levels than the control group. The Mann-Whitney U test (N = 47) revealed a significant difference in satisfaction levels between the "3-in-1" method and the control group (U = 379.5, p = 0.016), as shown in Figure 3.

Perceived Understanding of the Content

The "3-in-1" intervention group demonstrated significantly higher perceived mastery of biostatistics (7.74) than the control group (5.58), a difference of 2.16 points (p < 0.001). These results confirm the positive effect of active learning strategies on self-perception of learning, as shown in Table 3.

**Table 3:** Comparison of perceived mastery of biostatistics content between groups.

Group	N	Mean ± SD	t	Sig.	Mean difference	95% CI, lower	95% CI, upper
Control	24	5.58 ± 1.88	-4.309	0.000	-2.156	-3.163	-1.148
“3-in-1”	23	7.74 ± 1.51					

Ninth versus second semester

**Figure 4:**
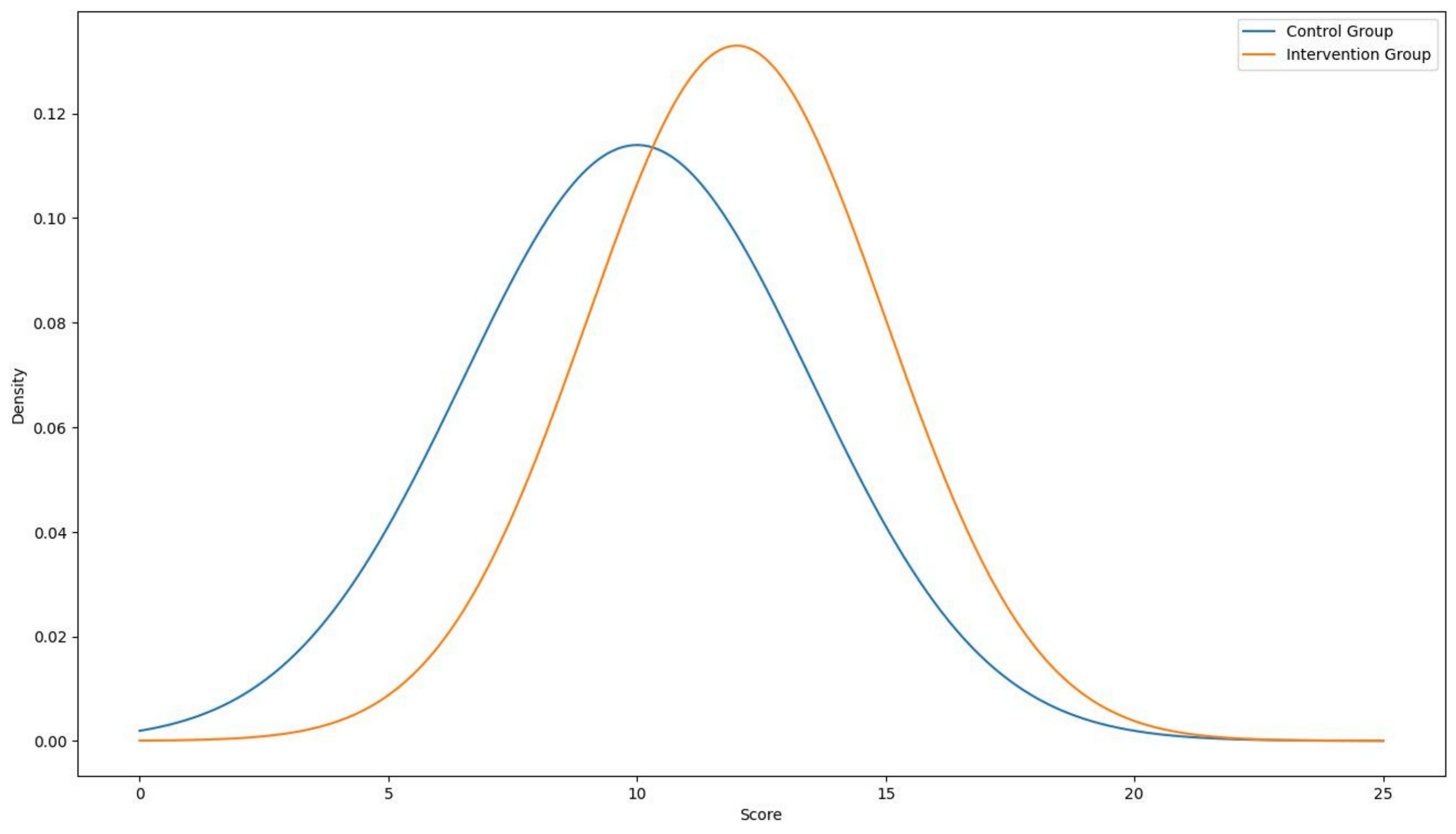
Comparison of scores by academic semester.

The analysis compared the scores of second-semester students (13.13 ± 3.07) with those of ninth-semester students (12.50 ± 2.68). Levene's test indicated homogeneity of variances (F = 0.085, p = 0.772), thus assuming equal variances in the t-test. The t-test for independent samples revealed no statistically significant differences between the groups (t = 0.664, p = 0.511), as shown in Figure 4. The mean difference was 0.63 points (95% CI: -1.29 to 2.55), indicating that the academic semester did not significantly influence the scores obtained.

The ‘3-in-1’ intervention group in the ninth semester had a perceived mastery score of 7.74 for biostatistics content, while the second-semester group had a satisfaction score of 7.58. There was no statistically significant difference between the two groups (p = 0.62), indicating comparable levels of perceived learning.

Satisfaction With the Teaching Method

**Figure 5:**
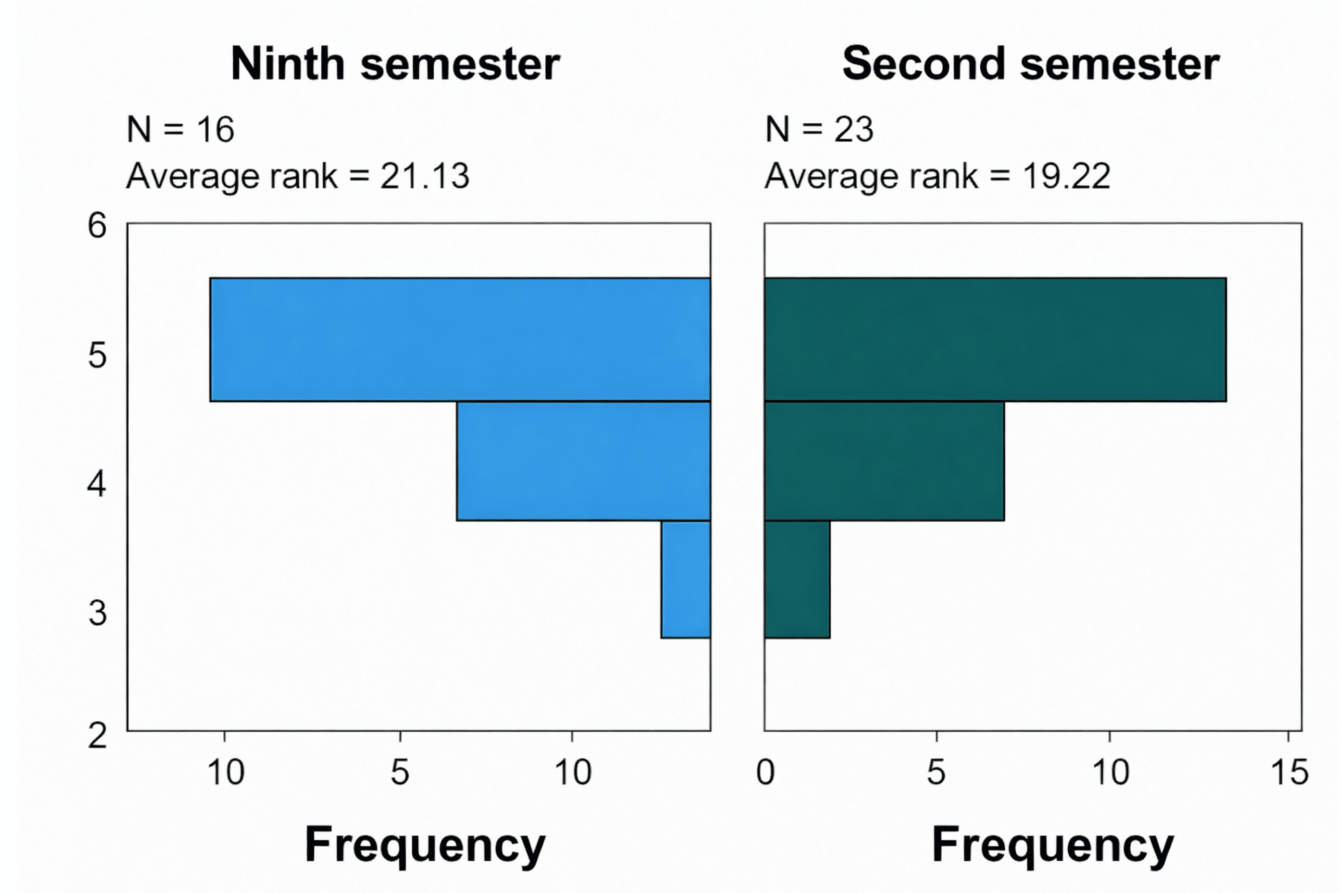
Satisfaction with the pedagogical method among second vs. ninth semester students.

The Mann-Whitney U test revealed no statistically significant differences in satisfaction with the teaching method between second- and ninth-semester students (U = 202.0; p = 0.526). This indicates that both groups have similar satisfaction levels. Figure 5 shows the difference in rank.

Perceived Understanding of the Content

The recoded score was dichotomized based on whether participants scored less than or equal to 11.2 points (70% of the total score). Inferential analysis revealed no statistically significant association between the academic semester and the recoded score. Pearson's chi-square test yielded a value of χ² = 1.02 with one degree of freedom (p = 0.312). From a risk estimation perspective, OR = 0.46 (95% CI: 0.10-2.10) indicated that attending the second semester was not significantly associated with a higher probability of receiving a score below 11.2 points compared to the ninth semester.

The ‘3-in-1’ intervention group in the ninth semester had a perceived mastery score of 7.74 for biostatistics content, while the second-semester group had a satisfaction score of 7.58. There was no statistically significant difference between the two groups (p = 0.62), indicating comparable levels of perceived learning.

## Discussion

Regarding results, the “3-in-1” intervention group achieved significantly higher knowledge test scores than the control group (mean = 13.13 vs. 10.92; p = 0.023), indicating a positive effect of the intervention. Satisfaction with the teaching method was also significantly higher in the “3-in-1” group (p = 0.016), as was perceived understanding of biostatistics content, with a mean difference of 2.16 points in favor of the intervention (p < 0.001). Comparisons by academic semester showed no significant differences between second- and ninth-semester students in knowledge scores, satisfaction, or perceived understanding, suggesting that the observed benefits were attributable to the teaching method rather than the level of academic progression.

These findings were consistent with those of previously published studies [4,16-20] that evaluated diverse pedagogical strategies for teaching biostatistics to medical students, particularly those emphasizing active, practical, and student-centered approaches over traditional lecture-based methods. Collectively, the evidence suggested that instructional models integrating application, interaction, and contextualization facilitated more effective learning outcomes in biostatistics education.

In this context, Hayes et al. [16] reported that a biostatistics module implemented among first- and second-year medical students, which integrated conceptual definitions, applied examples drawn from the medical literature, and formative assessments with immediate feedback, resulted in substantial improvements in objective biostatistics knowledge. Among students who completed the pre- and post tests (n = 32), mean knowledge scores increased from 2.41 to 3.53 (p < 0.001), and significant gains were observed in confidence selecting appropriate biostatistical tests, understanding their use in medical literature, and familiarity with biostatistics (p < 0.001). Students also reported high levels of satisfaction, highlighting the module’s applicability and ease of use, thereby supporting its effectiveness in strengthening biostatistical competencies during medical training.

Consistent with these results, another study comparing the teaching of biostatistics through problem-based learning (PBL) with the traditional lecture method demonstrated more favorable outcomes for the PBL approach [17]. Students taught through PBL obtained higher scores in objective (17.29 vs. 13.54; p = 0.03), descriptive (41.06 vs. 30.67; p = 0.01), viva voce (16.83 vs. 12.98; p = 0.034), and total assessment scores (75.18 vs. 57.19; p = 0.01). In addition, PBL was perceived as a more effective approach, as it promoted self-directed learning, integration of knowledge, and the development of critical thinking compared with traditional lecturing. These findings reinforced the notion that PBL represented a more effective instructional strategy within the framework of competency-based medical education.

In line with these pedagogical trends, an article describing the development and evaluation of a semester-long Biostatistical Literacy course at the University of Minnesota provided additional evidence supporting a conceptual and application-oriented approach to biostatistics education. This course was designed to train students to understand, interpret, and critically evaluate the statistical methods and results commonly used in medical and public health literature. Rather than emphasizing computational procedures, the curriculum focused on statistical reasoning, including the interpretation of confidence intervals, p-values, regression models, and survival analyses, as well as the critical reading of the methods and results sections of scientific articles. Implemented through a flipped-classroom and active-learning model, the course was completed by 1,276 students between 2014 and 2020, achieving a mean final assessment score of 75.1%, with comparable performance across online and in-person formats. Overall, the findings supported the effectiveness of a literacy-focused biostatistics curriculum in strengthening students’ confidence and competence in critically appraising biomedical research [18].

Similarly, a study analyzing the perceptions of 527 medical students from seven faculties in Poland regarding traditional versus practical biostatistics teaching methods found that the practical approach (based on the critical reading of scientific articles and the guided application of statistical tests) was associated with lower class- and exam-related stress and higher student satisfaction and perceived acquisition of practical knowledge (all p < 0.001), while traditional teaching was linked to greater anticipated difficulty in interpreting research findings (p < 0.001) [4]. Overall, the results supported the superiority of practical biostatistics instruction in improving student experience and perceived competence.

Importantly, the results of the present study indicated that the “3-in-1” pedagogical intervention had a positive and statistically significant impact on satisfaction with the teaching method and on perceived understanding of biostatistics among medical students. These findings were consistent with a quasi-experimental study conducted in Brazil in 2017 [19], which reported that the integration of R and RStudio into biostatistics instruction was associated with improvements in students’ attitudes toward statistics, perceived competence, and academic performance. Together, these results suggested that the incorporation of integrated, technology-supported, and practice-oriented strategies enhanced both cognitive and affective learning outcomes in biostatistics education.

Finally, the experience of a Research-Oriented Medical Education (ROME) program at the Institute of Medical Sciences in Pondicherry, India, provided additional support for these pedagogical trends. In this program, applied biostatistics instruction strengthened undergraduate medical students’ knowledge, skills, and confidence through structured training in research methodology, statistical analysis, graphical representation, and the use of software such as MS Excel and SPSS. This experience demonstrated that practical and systematic approaches to teaching biostatistics were essential not only for medical training but also for clinical practice and future academic development [20].

The lack of specialized statistical reviews in publications, the increased complexity of methods, time constraints in academia, and the economic burden of medical training all reinforce the need for accessible, efficient, and cost-effective educational resources to strengthen biostatistical skills in medical curricula [16].

Limitations

The main limitations of this study include its single-center design and relatively small sample size, which may limit the generalizability of the findings. In addition, a baseline age difference between groups could have influenced performance outcomes. The assessment was conducted immediately after the intervention, preventing evaluation of long-term knowledge retention. Furthermore, satisfaction and perceived mastery were measured using self-reported scales, which are prone to subjective bias, and the lack of blinding inherent to the educational intervention may have introduced expectancy effects.

## Conclusions

The active “3-in-1” approach proved to be effective in improving academic performance in biostatistics among medical students at UNIANDES, as evidenced by significantly higher knowledge test scores compared with the traditional teaching method. The “3-in-1” methodology had a positive impact on students’ attitudes and motivation toward learning biostatistics, reflected in higher levels of satisfaction and greater perceived understanding of the content.

Overall, the stratified design demonstrated that the “3-in-1” active learning approach is superior to the traditional methodology in key educational outcomes (academic performance, satisfaction, and self-perceived mastery), supporting its implementation in medical education.

## Appendices

**Table 4:** Original questionnaire developed for academic purposes in Biostatistics. Credits: Quinapanta Castro NI and Orbea Fernández A.

Original text (Spanish)	Translated text (English)
1. En un estudio epidemiológico se registró el número de cigarrillos fumados diariamente por 100 pacientes adultos. Desde el punto de vista bioestadístico, esta variable se clasifica como: a) Cualitativa nominal b) Cualitativa ordinal c) Cuantitativa discreta d) Cuantitativa continua	1. In an epidemiological study, the number of cigarettes smoked daily by 100 adult patients was recorded. From a biostatistical point of view, this variable is classified as: (a) Nominal qualitative, (b) ordinal qualitative, (c) discrete quantitative, and (d) continuous quantitative
2. En un estudio transversal se desea representar la distribución de frecuencias del grupo sanguíneo (A, B, AB, O) en una población. ¿Cuál es el gráfico más apropiado? a) Histograma b) Gráfico de barras c) Diagrama de dispersión d) Polígono de frecuencias	2. In a cross-sectional study, the frequency distribution of blood group (A, B, AB, O) in a population is to be represented. Which is the most appropriate graph? (a) Histogram, (b) bar chart, (c) scatter plot, and d) frequency polygon
3. En un análisis descriptivo de la presión arterial sistólica se calcularon varios estadísticos. ¿Cuál de los siguientes corresponde exclusivamente a una medida de dispersión? a) Media b) Mediana c) Desviación estándar d) Moda	3. In a descriptive analysis of systolic blood pressure, several statistics were calculated. Which of the following corresponds exclusively to a measure of dispersion? (a) Mean, (b) median, (c) standard deviation, and (d) mode
4. En un estudio sobre el índice de masa corporal (IMC) en adultos, ¿cuál de las siguientes opciones corresponde a una medida de tendencia central? a) Rango b) Varianza c) Media d) Desviación estándar	4. In a study on body mass index (BMI) in adults, which of the following options corresponds to a measure of central tendency? (a) Range, (b) variance, (c) mean, and (d) standard deviation
5. El valor p obtenido en una prueba de hipótesis se interpreta correctamente como: a) La probabilidad de que la hipótesis nula sea verdadera b) La probabilidad de obtener los resultados observados, o más extremos, si la hipótesis nula es verdadera c) El nivel de confianza del estudio d) El tamaño del efecto observado	5. The p-value obtained in a hypothesis test is correctly interpreted as: (a) The probability that the null hypothesis is true. (b) The probability of obtaining the observed results, or more extreme ones, if the null hypothesis is true. (c) The confidence level of the study. (d) The observed effect size
6. Un intervalo de confianza del 95% para una media poblacional indica: a) Que el 95% de los valores individuales se encuentran dentro del intervalo b) Un rango de valores dentro del cual se espera que se encuentre el parámetro poblacional con un 95% de confianza c) La probabilidad de cometer un error tipo I d) Que el resultado es estadísticamente significativo	6. A 95% confidence interval for a population mean indicates: (a) That 95% of individual values fall within the interval. (b) A range of values within which the population parameter is expected to lie, with 95% confidence. (c) The probability of committing a Type I error. (d) That the result is statistically significant
7. Desde una perspectiva metodológica correcta, el primer paso en un proceso bioestadístico aplicado a la investigación médica es: a) Análisis de datos b) Interpretación de resultados c) Recolección de datos d) Planteamiento del problema y definición de objetivos	7. From a correct methodological perspective, the first step in a biostatistical process applied to medical research is: (a) data analysis, (b) interpretation of results, (c) data collection, and (d) problem formulation and definition of objectives
8. Una vez que los datos han sido correctamente recolectados, el segundo paso del proceso bioestadístico consiste en: a) Interpretación clínica de los resultados b) Análisis estadístico de los datos c) Comunicación científica de los hallazgos d) Formulación de conclusiones	8. Once data have been properly collected, the second step in the biostatistical process consists of: (a) clinical interpretation of results, (b) statistical analysis of the data, (c) scientific communication of findings, and (d) formulation of conclusions
9. En un estudio clínico se comparan los niveles medios de hemoglobina entre dos grupos independientes de pacientes. ¿Qué prueba estadística es la más apropiada si los datos siguen una distribución normal y presentan varianzas homogéneas? a) Chi-cuadrado b) Prueba t de Student para muestras independientes c) ANOVA d) U de Mann-Whitney	9. In a clinical study, mean hemoglobin levels are compared between two independent groups of patients. Which statistical test is most appropriate if the data follow a normal distribution and have homogeneous variances? (a) Chi-square, (b) Student’s t-test for independent samples, (c) ANOVA, and (d) Mann-Whitney U test
10. En un estudio se comparan los valores medios de glucosa en ayunas (mg/dL) entre tres grupos independientes de pacientes. ¿Qué prueba estadística es la más apropiada bajo supuestos de normalidad? a) Prueba t de Student b) Chi-cuadrado c) ANOVA de una vía d) Kruskal-Wallis	10. In a study, mean fasting glucose values (mg/dL) are compared among three independent groups of patients. Which statistical test is most appropriate under the assumptions of normality? (a) Student’s t-test, (b) chi-square, (c) one-way ANOVA, and (d) Kruskal-Wallis
11. En un estudio se desea evaluar la asociación entre el sexo (masculino/femenino) y la presencia de hipertensión arterial (sí/no). ¿Qué prueba estadística es la más apropiada? a) Prueba t de Student b) ANOVA c) Chi-cuadrado d) Correlación de Pearson	11. In a study, the association between sex (male/female) and the presence of arterial hypertension (yes/no) is to be evaluated. Which statistical test is most appropriate? (a) Student’s t-test, (b) ANOVA, (c) chi-square, and (d) Pearson correlation
